# KPC-2-Producing Carbapenem-Resistant *Klebsiella pneumoniae* of the Uncommon ST29 Type Carrying OXA-926, a Novel Narrow-Spectrum OXA β-Lactamase

**DOI:** 10.3389/fmicb.2021.701513

**Published:** 2021-08-27

**Authors:** Lina Liu, Yu Feng, Li Wei, Yuling Xiao, Zhiyong Zong

**Affiliations:** ^1^Center for Pathogen Research, West China Hospital, Sichuan University, Chengdu, China; ^2^Department of Infection Control, West China Hospital, Sichuan University, Chengdu, China; ^3^Laboratory of Clinical Microbiology, Department of Laboratory Medicine, West China Hospital, Sichuan University, Chengdu, China; ^4^Center of Infectious Diseases, West China Hospital, Sichuan University, Chengdu, China; ^5^Division of Infectious Diseases, State Key Laboratory of Biotherapy, Chengdu, China

**Keywords:** *Klebsiella pneumoniae*, carbapenem resistance, β-lactamase, OXA, plasmid

## Abstract

We isolated and characterized a carbapenem-resistant *Klebsiella pneumoniae* (CRKP) clinical strain from blood carrying a novel *bla*_OXA_ gene, *bla*_OXA–926_, and belonging to ST29, an uncommon CRKP type. The strain, 130002, was genome sequenced using both short- and long-read sequencing and has a 94.9-kb self-transmissible IncFII plasmid carrying *bla*_KPC–2_. *K. pneumoniae* genomes of the ST29 complex (ST29 and its single-allele variants) were retrieved and were subjected to single nucleotide polymorphism-based phylogenomic analysis. A total of 157 genomes of the ST29 complex were identified. This complex is commonly associated with extended-spectrum β-lactamase-encoding genes, in particular, *bla*_CTX–M–15_ but rarely has carbapenemase genes. The novel plasmid-encoded β-lactamase-encoding gene *bla*_OXA–926_ was identified on a 117.8-kb IncFIA-IncFII plasmid, which was transferrable in the presence of the *bla*_KPC–2_-carrying plasmid. *bla*_OXA–926_ was cloned and MICs of β-lactams in the transformants were determined using microdilution. OXA-926 has a narrow spectrum conferring reduced susceptibility only to piperacillin, piperacillin-tazobactam, and cephalothin. Avibactam cannot fully inhibit OXA-926. *bla*_OXA–926_ and its variants have been seen in *Klebsiella* strains in Asia and Brazil. OXA-926 is the closest in sequence identity (89.9%) to a chromosome-encoding OXA-type enzyme of *Variovorax guangxiensis*. In conclusion, OXA-926 is novel plasmid-borne narrow-spectrum β-lactamase that cannot be fully inhibited by avibactam. It is likely that *bla*_OXA–926_ originates from a species closely related to *V*. *guangxiensis* and was introduced into *Klebsiella* > 10 years ago.

## Introduction

Resistance to β-lactam agents such as penicillins, cephalosporins, and carbapenems in the *Enterobacteriaceae*, one of the most common human pathogens, is mainly due to the production of hydrolyzing enzymes called β-lactamases. β-Lactamases can be divided into classes A, B, C, and D based on amino acid homology (Hall and Barlow, 2005). Class A, C, and D enzymes are also termed serine β-lactamases as they possess a serine at the active site, while class B enzymes require a metal ion for activity and are therefore called metallo-β-lactamases. OXA (oxacillinase) is a large group of class D β-lactamases with a remarkably varied spectrum against β-lactam agents from narrow-spectrum (hydrolyzing penicillins only, e.g., OXA-1) to extended-spectrum (with ability to hydrolyze 3rd generation cephalosporins, e.g., OXA-11) and carbapenemases (e.g., OXA-23 and OXA-48) (Evans and Amyes, 2014). A number of bacterial species, e.g., *Acinetobacter baumannii* and *Pseudomonas aeruginosa* contain intrinsic OXA-encoding genes *bla*_OXA_ in their chromosomes, while many *bla*_OXA_ genes are carried by plasmids (Evans and Amyes, 2014). In this study, we found a *bla*_OXA_ gene encoding a novel OXA enzyme in a carbapenem-resistant *Klebsiella pneumoniae* (CRKP) clinical strain and determined its active spectrum. We also found that this strain belongs to ST29, an uncommon CRKP type. CRKP has emerged worldwide as a significant human health challenge (World Health Organization, 2017). The global dissemination of CRKP is mainly due to certain high-risk clones, in particular, ST 258 (Adler et al., 2014) and ST11 in China (Qi et al., 2011), but new CRKP lineages are continuously emerging. We, therefore, analyzed all available genomes of ST29 and found that this ST is commonly associated with the carriage of extended-spectrum β-lactamase-encoding genes rather than carbapenemases genes.

## Materials and Methods

### The Study, the Strain, and Susceptibility Testing

Strain 130002 was recovered from the blood of an ICU patient in 2020 at West China Hospital as part of routine care. MICs of aztreonam, ceftazidime, ceftazidime-avibactam, cefepime, ertapenem, imipenem, meropenem, piperacillin-tazobactam, and colistin were determined using the broth microdilution method of the Clinical and Laboratory Standards Institute (CLSI) (CLSI, 2020). This study has been approved by the Ethical Committee of West China Hospital without the requirement of an informed consent due to the fact that no patient information is needed.

### Short- and Long-Read Whole Genome Sequencing and Analysis

Strain 130002 was subjected to whole genome sequencing using both a HiSeq X10 sequencer (Illumina; San Diego, CA, United States; 200×) and a MinION Sequencer (Nanopore; Oxford, United Kingdom). Genomic DNA was prepared using the QIAamp DNA Mini Kit (Qiagen, Hilden, Germany). Both short (Illumina) and long (Nanopore) reads were utilized to generate a *de novo* hybrid assembly using Unicycler (Wick et al., 2017) and Pilon (Walker et al., 2014). Sequence type (ST) was determined by querying the multilocus sequence typing database^1^, while capsule (KL) typing was performed using Kleborate (Wyres et al., 2016). Antimicrobial resistance genes were identified from the genome sequences using the ABRicate program^2^ to query the ResFinder database^3^. Replicon sequence types of IncF plasmids were determined using the pMLST^4^. Plasmid comparison was performed using BRIG (Alikhan et al., 2011) in the default settings. Insertion sequences were identified using ISFinder^5^.

### Conjugation

Mating experiments were performed in broth and on filters with *Escherichia coli* J53 AizR (an azide resistant variant of J53) as the recipient at both 25 and 37°C, as described previously (Coque et al., 2002). Potential transconjugants were selected on LB agar plates containing 16/4 mg/L of piperacillin-tazobactam and 150 mg/L of sodium azide. The presence of *bla*_KPC–2_ and *bla*_OXA–926_ in the transconjugants was confirmed by PCR with primers KPC-up1/KPC-dw1 (5′-CCTA GCTCCACCTTCAAACAA/GTGAGGGCGAAGGTTAAATG) (Zhang et al., 2012) and OXA926-Fw/OXA926-Rev (see below), respectively, and subsequent Sanger sequencing.

### Cloning of *bla*_OXA–926_ and Function Characterization

To determine the activity of OXA-926, the 807-bp complete coding sequence of *bla*_OXA–926_ was amplified from strain 130002 using primers OXA926-Fw/Rev (5′-CCGGATCCATGTGCA ATCGCATCCTCCA/CCCTCGAGTCAATGGTCGATGGCTGG CA; restriction sites are underlined). PCR amplicons and the vector pET-28a (Fenghbio; Changsha, China) were digested using *BamH*I and *Xho*I (New England Biolabs, Ipswich, MA, United States) and were then ligated to the pET-28a vector using T4 ligase (New England Biolabs) to construct pET28a-OXA926. The constructed plasmid was transformed into *E. coli* strain BL21 by chemical, as described before (Sambrook and Russell, 2001). Potential transformants containing pET28a-OXA926 were selected on Luria–Bertani agar plates (Sigma; St. Louis, MO, United States) containing 50 mg/L of kanamycin. Colonies on plates were screened for *bla*_OXA–926_ by PCR using primers OXA926-Fw/Rev and subsequent Sanger sequencing. The empty vector pET-28a was also transformed into BL21 for control.

MICs of aztreonam, ampicillin, ampicillin-sulbactam, piperacillin, piperacillin-tazobactam, oxacillin, cefazolin, cephalothin, cefuroxime, ceftriaxone, cefotaxime, ceftazidime, ceftazidime-avibactam, cefepime, cefoxitin, ertapenem, imipenem, and meropenem for the transformant containing pET28a-OXA926 (BL21::pET28a-OXA926) were determined as described above. MICs of piperacillin in the presence of 4 mg/L of avibactam were also determined based on the methods to determine MICs of ceftazidime-avibactam (CLSI, 2020).

### Protein Analysis

The secondary structure of OXA-926 β-lactamase was predicted using the neural network based web service JPred4 (Drozdetskiy et al., 2015) with the default settings. The origin of OXA-926 was investigated using BlastP^6^.

### Phylogenomic Analysis of the ST29 Complex

All complete and draft genomes of *K. pneumoniae* belonging to the ST29 complex including ST29 and its single-allele variants, i.e., ST193, 465, 711, 723, 985, 1161, and 1271 (the allele profile of these STs is shown in Table 1) were retrieved from GenBank including the SRA database (accessed during May 2020). The genome sequences were mapped against the complete genome of 130002 for single nucleotide polymorphisms (SNP) calling using Snippy v4.6.0^7^ with the default settings. Gubbins v2.4.1 (Croucher et al., 2015) was used for recombination filtering prior to the phylogenomic reconstruction using RAxML v8.2.12 (Stamatakis, 2014) under the GTRGAMMA model and a 1,000-bootstrap test. Trees were annotated and viewed in iTOL v5.7 (Letunic and Bork, 2019) and FigTree v1.4.4^8^. As there are up to 1,048 SNPs between the genomes of ST29 and its single-allele variants (see below), suggesting a significant phylogenetic divergence, we, therefore, did not include the double-allele variants of ST29 for analysis.

**TABLE 1:**
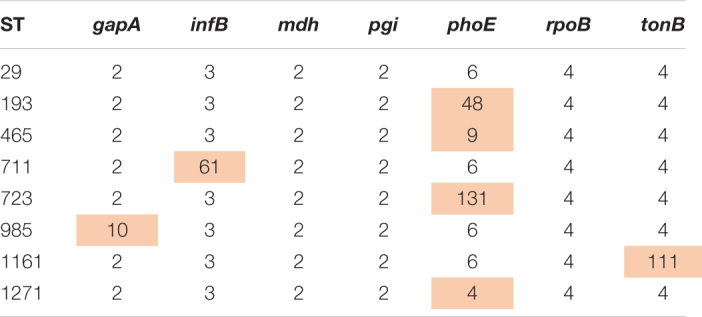
The allele profile of ST29 and its single-allele variants.

*The allele differences are highlighted.*

### Retrieval of *bla*_OXA–926_-Carrying Strains From GenBank

Draft and complete genome sequences deposited in GenBank were screened by BlastN (see text footnote 6) for the presence of *bla*_OXA–926_. Metadata of *bla*_OXA–926_-carrying strains including host species and countries and year of isolation were retrieved.

## Results and Discussion

### CRKP Strain 130002 Belongs to ST29, an Uncommon CRKP Type

Strain 130002 was resistant to aztreonam, ceftazidime, cefepime, ertapenem, imipenem, meropenem, and piperacillin-tazobactam but was susceptible to ceftazidime-avibactam and was intermediate to colistin (Table 2). The complete genome sequence of strain 130002 was obtained by *de novo* hybrid assembly of both short (Illumina) and long (Nanopore) reads and had a 5.3-Mb circular chromosome with three plasmids (Table 3). Strain 130002 belongs to ST29, an uncommon CRKP type, and the KL62 capsule type.

**TABLE 2:** MIC (mg/L) of β-lactams for 130002 and *E. coli* BL21 expressing OXA-926 or not.

	**130002**	**BL21::pET28a-OXA926**	**BL21::pET28a**
Aztreonam	>256	0.03	0.03
Ampicillin	–	1	1
Ampicillin-sulbactam	–	1/0.5	0.5/0.25
Piperacillin	>256	32	0.5
Piperacillin-tazobactam	>256/4	16/4	1/4
Piperacillin-avibactam	8/4	4/4	0.5/4
Oxacillin	–	512	512
Cefazolin	–	0.5	0.5
Cephalothin	–	4	0.25
Cefuroxime	–	0.25	0.25
Ceftriaxone	–	0.03	0.03
Cefotaxime	–	≤0.015	≤0.015
Ceftazidime	32	0.06	0.06
Ceftazidime-avibactam	0.5/4	0.06/4	0.06/4
Cefepime	16	0.03	0.03
Cefoxitin	–	1	1
Ertapenem	>256	0.03	0.03
Imipenem	64	0.5	0.5
Meropenem	128	0.06	0.06
Colistin	1	–	–

**TABLE 3:** The complete genome and antimicrobial resistance genes of isolate 013002.

	**Accession no.**	**Size, bp**	**Replicon type**	**Genes mediating resistance to**					
				**β -lactam**	**Aminoglycoside**	**Quinolone**	**Fosfomycin**	**Sulfonamide**	**Tetracycline**
Chromosome	CP064851	5,297,461	–	*bla*_SHV–187_		*oqxA-B*	*fosA6*		*tet(34)*
p1_130002	CP064854	164,362	IncC2		*ant(2”)-Ia*	*qnrA1*		*sul1*	
pKPC2_130002	CP064852	94,968	IncFII(Yp)_1_Yersenia	*bla*_KPC–2_					
pOXA926_130002	CP064853	117,839	IncFIA(HI1)_1_HI1, IncFII_1_pKP91	*bla*_OXA–926_					

### The ST29 Complex of *K. pneumoniae* Is Widely Distributed and Commonly Associated With *bla*_CTX–M_ Genes but Rarely With Carbapenemase Genes

**FIGURE 1:**
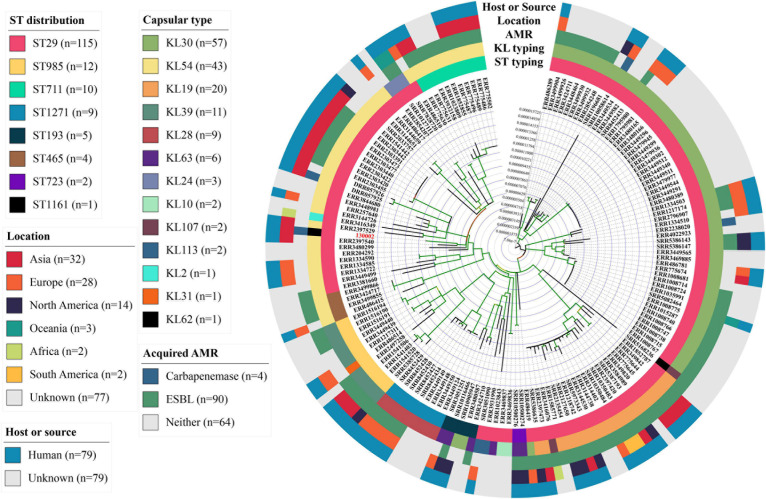
Phylogenomic tree of *K. pneumoniae* strains of the ST29 complex. This phylogenomic tree of 130002 and 157 genomes of the ST29 complex is based on SNP calling using Snippy v4.6.0 and filtering recombination using Gubbins v2.4.1. The phylogeny was inferred using RAxML v8.2.12 under the GTRGAMMA model with a 1,000-bootstrap test and is annotated and viewed in iTOL v5.7 and FigTree v1.4.4. More information of the genomes is provided in Supplementary Datasheet 1.

As ST29 is an uncommon type of CRKP, we retrieved all genomes of *K. pneumoniae* belonging to the ST29 complex including ST29 and its single-allele variants (ST193, 465, 711, 723, 985, 1161, and 1271) from GenBank. A total of 157 genomes were identified and strains of the ST29 complex have been identified in all continents but Antarctica (Supplementary Datasheet 1). Most strains (57.3%, 90/157) of the ST29 complex carried genes encoding extended-spectrum β-lactamases (ESBL), in particular, *bla*_CTX–M–15_ (*n* = 69), while only four strains had carbapenemase genes (*bla*_KPC–2_, *bla*_KPC–3_, or *bla*_IMP–19_; Supplementary Datasheet 1). Strains of the ST29 complex were assigned to 13 capsular types (KL2, 10, 19, 24, 28, 30, 31, 33, 54, 62, 63, 107, and 113; Figure 1), while 130002 is the only strain of KL62. There was a maximum of 1,048 SNPs between strains of the ST29 complex (Supplementary Datasheet 2), suggesting that the complex is diverse in clonal background. Strain 130002 had a range of 258–904 SNPs with other strains of the ST29 complex (Supplementary Datasheet 2) and is most closely related to ST29 KL54 strain 4300STDY6470438 (accession no. ERR2397540) recovered from an unspecified human sample in Thailand in 2016 (Figure 1).

### 130002 Has a *bla*_KPC–2_ and *bla*_OXA_ Gene Encoding a Novel Narrow-Spectrum β-Lactamase OXA-926

**FIGURE 2:**
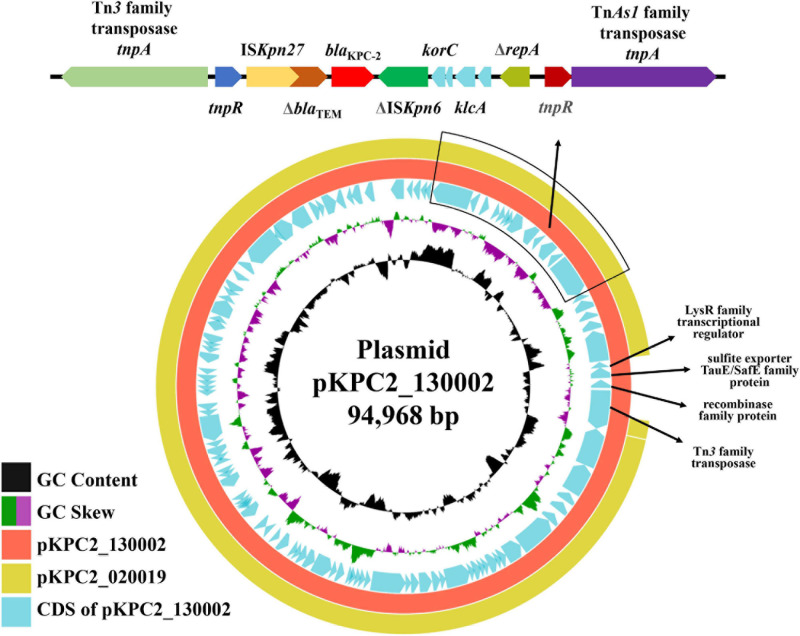
Alignment of pKPC2_130002 with pKPC2_020019. pKPC2_130002 is a 94.9-kb IncFII plasmid of the Y6:A-:B- type and has *bla*_KPC–2_. pKPC2_130002 is the closest to pKPC2_020019 (accession no. CP028554), an 89.7-kb Y6:A-:B-type *bla*_KPC–2_-carrying plasmid from a *K. variicola* strain recovered in a hospital of a neighbor city (Meishan, 80 km away from Chengdu) in 2017, with a 94% coverage and 99.93% identity. Comparing with pKPC2_130002, pKPC2_020019 lacks several protein-encoding genes with the products being indicated. On pKPC2_130002 and pKPC2_020019, *bla*_KPC–2_ is located in a non-Tn*4401* element containing a transposase-encoding *tnpA* gene of an unnamed transposon of the Tn*3* family at upstream and another *tnpA* of an unnamed transposon of the Tn*As1* family at downstream. This comparison was performed using BRIG (Alikhan et al., 2011) in the default settings.

Strain 130002 contains three β-lactamase-encoding genes including narrow-spectrum β-lactamase gene *bla*_SHV–187_ (Tian et al., 2020) on chromosome, carbapenemase gene *bla*_KPC–2_ on a 94.9-kb IncFII plasmid of the Y6:A-:B- type (designated pKPC2_130002), and a novel *bla*_OXA_ gene on a 117.8-kb plasmid containing both IncFIA and IncFII replicons (designated pOXA926_130002; Table 3). pKPC2_130002 shows the closest similarity among the sequenced plasmids to pKPC2_020019 (accession no. CP028554), an 89.7-kb Y6:A-:B-type *bla*_KPC–2_-carrying plasmid from a *Klebsiella variicola* strain recovered from a hospital of a neighboring city (Meishan, 80 km away from Chengdu) in 2017, with a 94% coverage and 99.93% identity (Figure 2). On pKPC2_130002 and pKPC2_020019, *bla*_KPC–2_ is located in a non-Tn*4401* element containing a transposase-encoding *tnpA* gene of an unnamed transposon of the Tn*3* family at upstream and another *tnpA* of an unnamed transposon of the Tn*As1* family at downstream (Figure 2). Transconjugants containing *bla*_KPC–2_ were obtained at a frequency of 1 × 10^–4^ (transconjugant per recipient), illustrating that pKPC2_130002 is readily self-transmissible. The findings above show that strain 130002 emerged as a CRKP by acquiring a self-transmissible *bla*_KPC–2_-carrying plasmid.

**FIGURE 3:**
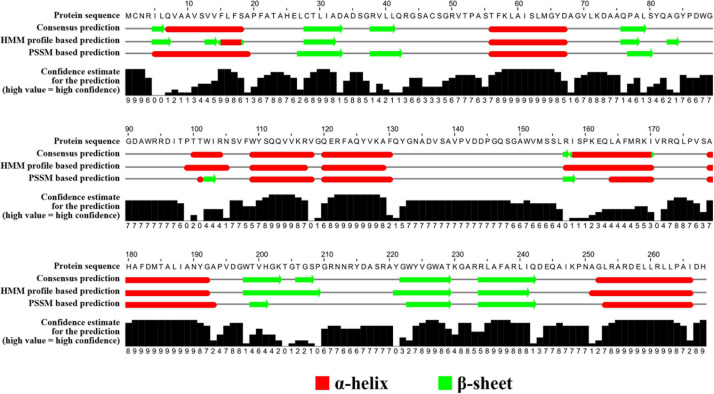
Secondary structure of OXA-926. The secondary structure was predicted using the neural network-based web service JPred4 (Drozdetskiy et al., 2015) with the default settings. Secondary structure elements, α helixes, β sheets, and 3_10_-helixes (representing by η), are indicated. β-strands are rendered as arrows, and strict α- and β-turns are shown as TTT and TT letters, respectively.

The *bla*_OXA_ gene encodes an OXA enzyme that shows the closest similarity to OXA-459 with a 59.1% amino acid (aa) identity (143/242 aa) and 90.3% coverage (242/268 aa) among all known OXA enzymes in the Bacterial Antimicrobial Resistance Reference Gene Database^9^. OXA-459 is one of the OXA-114-like enzymes intrinsic to *Achromobacter* spp. The findings above suggest that this OXA is a novel enzyme and is assigned OXA-926 by the Pathogen Detection group of GenBank, National Center for Biotechnology Information. OXA enzymes are very diverse in amino acid sequences and can be assigned to various subfamilies (Evans and Amyes, 2014; Yoon and Jeong, 2021). A ≥73.1% amino acid identity has been recently proposed as the cutoff to define OXA subfamilies (Yoon and Jeong, 2021) and, therefore, OXA-926 represents a novel subfamily. The secondary structure of OXA-926 contains seven α helixes, six β sheets, and four 3_10_-helixes (Figure 3).

*bla*_OXA–926_ was successfully cloned into vector pET28a, generating pET28a-OXA926. Among the β-lactams tested, only the MICs of piperacillin, piperacillin-tazobactam, and cephalothin for the transformant containing pET28a-OXA926 (BL21::pET28a-OXA926) were increased by ≥four-fold as compared with those for the transformant containing pET-28a (BL21::pET28a) (Table 2). This suggests that OXA-926 exhibits activity against piperacillin and such activities cannot be inhibited by tazobactam, a class A (not class D) β-lactamase inhibitor. As avibactam is a non-β-lactam β-lactamase inhibitor able to inhibit classes A, C, and D β-lactamases, we, therefore, determined the MICs of piperacillin in the presence of 4 mg/L of avibactam. In the presence of avibactam, MIC of piperacillin decreased from 32 to 4 mg/L for BL21::pET28a-OXA926 but was still 8-fold of that for BL21::pET28a (0.5 mg/L). This suggests that avibactam is able to provide protection for β-lactams from the hydrolysis OXA-926 to a certain extent but cannot fully inhibit OXA-926.

**FIGURE 4:**
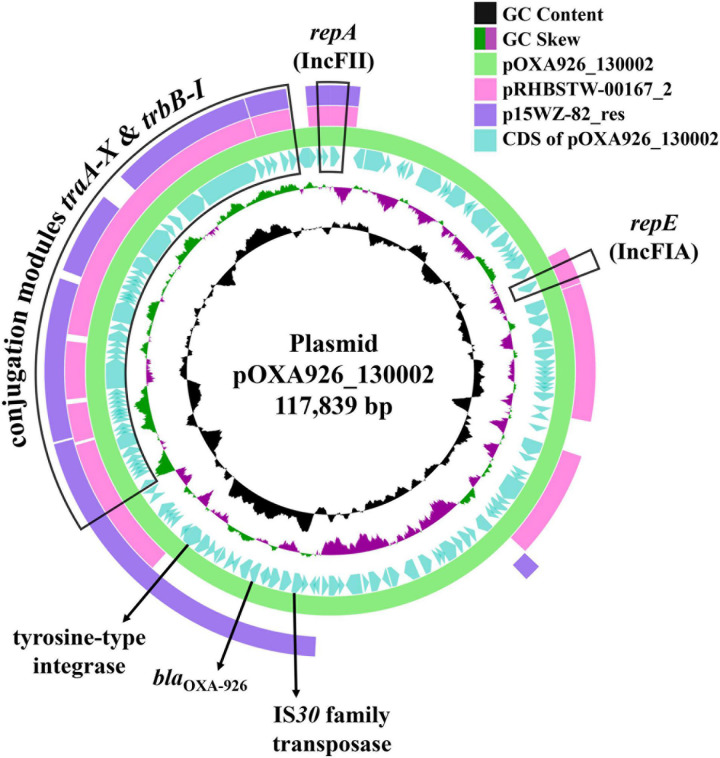
Alignment of pOXA926_130002 with RHBSTW-00167_2 and p15WZ-82_res. pOXA926_130002 is a 117.8-kb IncF plasmid and has *bla*_OXA–926_. Plasmid replication initiation-encoding genes *repA* of the IncFII replicon and *repE* of the IncFIA replicon and the conjugation module containing multiple genes (*traA* to *traX* and *trbB* to *trbI*) are indicated. A gene encoding the transpose of an IS*30* family insertion sequence at upstream of *bla*_OXA–926_ and a gene encoding a tyrosine recombinase-like integrase at downstream are also shown. pOXA926_130002 is the closest (56% coverage and 97.1% identity) to pRHBSTW-00167_2 (accession no. CP058119), a 223.8-kb K4:A10:B- type IncF plasmid with an additional IncR replicon of an *K. michiganensis* strain recovered in United Kingdom in 2017. pRHBSTW-00167_2 has no *bla*_OXA–926_, while p15WZ-82_res (accession no. CP032357) is another *bla*_OXA–926_-carrying plasmid of a *K. variicola* clinical strain recovered from an unspecified place in China in 2015. p15WZ-82_res belongs to the K4:A-:B- IncF type and also contains a K (pCAV1099-114 type) IncFIB allele. pOXA926_130002 had a 53% coverage and 99.0% identity with p15WZ-82_res.

pOXA926_130002 has a K4 IncFII allele and a novel IncFIA allele closest to the FIA_10 type with a 98.7% identity (378/383 nucleotides). pOXA926_130002 was the closest, with a 56% coverage and 97.1% identity (Figure 4), to pRHBSTW-00167_2 [accession no. CP058119; containing a K4 IncFII allele and a FIA_10 type IncFIA allele (K4:A10:B-) plus an IncR replicon] of *Klebsiella michiganensis* strain RHBSTW-00167 recovered from freshwater in the United Kingdom in 2017. By Blast, there is only one *bla*_OXA–926_-carrying plasmid, p15WZ-82_res (accession no. CP032357), with a complete sequence available in GenBank (Table 4). p15WZ-82_res was recovered from a *K. variicola* clinical strain in an unspecified place in China in 2015. This plasmid contains a K4 IncFII allele and a K (pCAV1099-114 type) IncFIB allele but no IncFIA allele (K4:A-:B-; pCAV1099-114 type IncFIB was not included in the IncF pMLST scheme). pOXA926_130002 had a 53% coverage and 99.0% identity with p15WZ-82_res (Figure 4). Transconjugants containing both *bla*_KPC–2_ and *bla*_OXA–926_ were obtained but those containing *bla*_OXA–926_ alone were not, suggesting that pOXA926_130002 was not self-transmissible. Nonetheless, pOXA926_130002 was able to be transferred in the presence of pKPC2_130002 at a frequency of 1 × 10^–6^ (transconjugant per recipient). On pOXA926_130002, there are no mobile genetic elements including insertion sequences and transposons present in the immediate upstream and downstream of *bla*_OXA–926_. Nonetheless, an unnamed novel insertion sequence of the IS*30* family was found 3,137 bp upstream of *bla*_OXA–926_ but no additional insertion sequences of the IS*30* family to form a composite transposon were present on pOXA926_130002 (Figure 4). A tyrosine recombinase-like integrase-encoding gene was present 4,984 bp downstream of *bla*_OXA–926_ (Figure 4) but no crossover sites (recombination sites), which are required for recombination mediated by this type of integrases are found. No additional tyrosine recombinase-like integrase-encoding gene was present upstream of *bla*_OXA–926_. Therefore, the mechanism for mobilizing *bla*_OXA–926_ remains to be elucidated and warrants further study.

**TABLE 4:** Genomes containing *bla*_OXA–926_ or *bla*_OXA–926_-like genes in GenBank^*a*^.

**Isolate**	**OXA**	**Species**	**aa identity, %**	**Accession no.**	**Country**	**Collection year**	**Host: sample type**
TUM14060	OXA-926	*Klebsiella pneumoniae*	100	BIIN00000000	Japan	2013	Human :–
4300STDY6470463	OXA-926	*Klebsiella pneumoniae*	100	UFFP00000000	Thailand	2016	Human: –
4300STDY6470462	OXA-926	*Klebsiella pneumoniae*	100	UFFK00000000	Thailand	2016	Human: –
4300STDY6470402	OXA-926	*Klebsiella pneumoniae*	100	UFDU00000000	Thailand	2016	Human: –
15WZ-82	OXA-926	*Klebsiella variicola*	100	CP032357 ^*c*^	China	2015	Human: –
K022	OXA-926	*Klebsiella variicola*	100	JACNNG000000000	China	2008	Human: –
TUM14096	OXA-926	*Klebsiella variicola*	100	BIJX00000000	Japan	2014	Human: –
ZKP186	OXA-926	*Klebsiella variicola*	100	CABWXA000000000	China	2017	Human: –
R8A	OXA-926-like^*b*^	*Klebsiella michiganensis*	97.02	JNCH00000000	Malaysia	2013	Human: dental plaque
Kv104	OXA-926-like^*b*^	*Klebsiella variicola*	97.02	JAAQPW000000000	Brazil	2017	Human: blood
Kv97	OXA-926-like^*b*^	*Klebsiella variicola*	97.02	JAAQPV000000000	Brazil	2017	Human: urine

*^*a*^*bla*_OXA–926_-like genes refer to those with ≥90% nucleotide identity with *bla*_OXA–926_.*

*^*b*^The three OXA-926-like are identical in aa sequence.*

*^*c*^In strain 15WZ-82, *bla*_OXA–926_ is carried on a plasmid, p15WZ-82_res, containing a K4 IncFII allele and a K (pCAV1099-114) IncFIB allele but no IncFIA allele.*

### *bla*_OXA–926_ May Originate From a Species Closely Related to *Variovorax*

BlastP shows that OXA-926 is the closest to a chromosome-encoding OXA-type enzyme of *Variovorax guangxiensis* (accession no. WP_184634888) with a 100% coverage and 89.9% (241/268) aa identity and is also similar to another chromosome-encoding OXA-type enzyme of *Variovorax gossypii* (accession no. WP_126469733) with a 98.9% (265/268) coverage and 85.4% (229/268) aa identity. *Variovorax* is a genus of the family *Comamonadaceae* within the order *Burkholderiales* (Taxonomy ID 34072 in NCBI). This suggests that *bla*_OXA–926_ originates from a yet unknown species likely within the genus *Variovorax*.

### *bla*_OXA–926_ Has Been Present in *Klebsiella* for More Than 10 Years

In GenBank, *bla*_OXA–926_ was found in one plasmid of *Klebsiella variicola* (GenBank accession no. CP032357) and seven *Klebsiella* draft genomes, including four *K. pneumoniae* and three *K. variicola* (Table 4). Four of the eight *Klebsiella* strains have detailed information available, revealing that the strains were recovered from China and Japan as far back as 2008. In addition, a variant of *bla*_OXA–926_ encoding an OXA enzyme with 97.01% (260/268) aa identity with OXA-926 was found in one *Klebsiella michiganensis* from Malaysia and two *K. variicola* from Brazil (Table 4). These findings suggest that *bla*_OXA–926_ has been circulating in *Klebsiella* spp. for more than a decade and has spread to multiple countries.

## Conclusion

We found a CRKP strain of an uncommon sequence and capsular type. Carbapenem resistance of this strain was due to the acquisition of a self-transmissible plasmid carrying *bla*_KPC–2_. We also found a novel plasmid-borne narrow-spectrum β-lactamase-encoding gene, *bla*_OXA–926_, able to confer a reduced susceptibility to piperacillin and piperacillin-tazobactam which cannot be fully inhibited by avibactam. It is likely that *bla*_OXA–926_ originates from a yet unknown species within the genus *Variovorax* of the order *Burkholderiales*.

## Data Availability Statement

The complete coding sequence of *bla*_OXA–926_ has been deposited into GenBank under accession no. MT767688. The complete sequence of the chromosome and plasmids of strain 130002 has been deposited into GenBank under the accession no. CP064851-CP064854.

## Author Contributions

ZZ designed the study. LL, LW, and YX performed the experiments. LL, YF, and ZZ performed the analysis. LL, YF, and ZZ drafted the manuscript. All authors contributed to the article and approved the submitted version.

## Conflict of Interest

The authors declare that the research was conducted in the absence of any commercial or financial relationships that could be construed as a potential conflict of interest.

## Publisher’s Note

All claims expressed in this article are solely those of the authors and do not necessarily represent those of their affiliated organizations, or those of the publisher, the editors and the reviewers. Any product that may be evaluated in this article, or claim that may be made by its manufacturer, is not guaranteed or endorsed by the publisher.

## Footnotes

1
http://bigsdb.pasteur.fr/klebsiella/klebsiella.html

2
https://github.com/tseemann/abricate

3
https://cge.cbs.dtu.dk/services/ResFinder/

4
https://cge.cbs.dtu.dk/services/pMLST/

5
https://www-is.biotoul.fr/

6
https://blast.ncbi.nlm.nih.gov/blast.cgi

7
https://github.com/tseemann/snippy

8
https://github.com/rambaut/figtree

9
https://www.ncbi.nlm.nih.gov/bioproject/PRJNA313047
